# LyeTxI-b, a Synthetic Peptide Derived From *Lycosa erythrognatha* Spider Venom, Shows Potent Antibiotic Activity *in Vitro* and *in Vivo*

**DOI:** 10.3389/fmicb.2018.00667

**Published:** 2018-04-06

**Authors:** Pablo V. M. Reis, Daiane Boff, Rodrigo M. Verly, Marcella N. Melo-Braga, María E. Cortés, Daniel M. Santos, Adriano M. de C. Pimenta, Flávio A. Amaral, Jarbas M. Resende, Maria E. de Lima

**Affiliations:** ^1^Departamento de Bioquímica e Imunologia, Instituto de Ciências Biológicas, Universidade Federal de Minas Gerais, Belo Horizonte, Brazil; ^2^Departamento de Química, Faculdade de Ciências Exatas, Universidade Federal dos Vales do Jequitinhonha e Mucuri, Diamantina, Brazil; ^3^Departamento de Odontologia Restauradora, Faculdade de Odontologia, Universidade Federal de Minas Gerais, Belo Horizonte, Brazil; ^4^Serviço de Proteômica e Aracnídeos – Diretoria de Pesquisa e Desenvolvimento, Fundação Ezequiel Dias, Belo Horizonte, Brazil; ^5^Departamento de Química, Instituto de Ciências Exatas, Universidade Federal de Minas Gerais, Belo Horizonte, Brazil

**Keywords:** LyeTxI, *Lycosa erythrognatha*, LyeTxI-b, antimicrobial peptide, septic arthritis

## Abstract

The antimicrobial peptide LyeTxI isolated from the venom of the spider *Lycosa erythrognatha* is a potential model to develop new antibiotics against bacteria and fungi. In this work, we studied a peptide derived from LyeTxI, named LyeTxI-b, and characterized its structural profile and its *in vitro* and *in vivo* antimicrobial activities. Compared to LyeTxI, LyeTxI-b has an acetylated N-terminal and a deletion of a His residue, as structural modifications. The secondary structure of LyeTxI-b is a well-defined helical segment, from the second amino acid to the amidated C-terminal, with no clear partition between hydrophobic and hydrophilic faces. Moreover, LyeTxI-b shows a potent antimicrobial activity against Gram-positive and Gram-negative planktonic bacteria, being 10-fold more active than the native peptide against *Escherichia coli.* LyeTxI-b was also active in an *in vivo* model of septic arthritis, reducing the number of bacteria load, the migration of immune cells, the level of IL-1β cytokine and CXCL1 chemokine, as well as preventing cartilage damage. Our results show that LyeTxI-b is a potential therapeutic model for the development of new antibiotics against Gram-positive and Gram-negative bacteria.

## Introduction

Super-resistant bacteria are an emerging public health problem. The urge to develop new tools and strategies to combat the superbugs is clear and was vehemently warned by the World Health Organization in a report about the global threat of antimicrobial resistance, in June 2014 (WHO, 2014). In a majority, this problem is associated with an evolutionary adaptation of bacteria that are found in a multicellular specialized formation called biofilm, which increases the resistance to conventional antibiotics (de la Fuente-Nunez et al., 2013, 2016). Thus, biofilm represents a challenge to treat. In the meantime, AMPs have become a new hope as tools against superbugs, as they are the primary immune defense of animals and can also be found in bacteria, fungi, and plants (Bulet et al., 1999; Ageitos et al., 2017). Although AMPs are less likely to induce resistance in bacteria, there are several studies demonstrating that these molecules induce resistance by multiple mechanisms, as increasing expression of proteases that can cleave the peptide, modifying plasma membrane composition and pump efflux (Nizet, 2006; Andersson et al., 2016). However, recently it has been shown that combination of different phylogenetic taxa AMPs, decrease the evolution of microbial resistance against them (Dobson et al., 2013). In addition, AMPs and derived peptides have shown activity against infections due to biofilm formation (de la Fuente-Nunez et al., 2016).

Antimicrobial peptides are typically composed of 7–44 amino acid residues, usually exhibiting amphipathic alpha helix or beta-sheet conformations and being positively charged (Jenssen et al., 2006; Mookherjee and Hancock, 2007; Dawson and Liu, 2008; Epand and Epand, 2011; Wang et al., 2012), although other structural arrangements such as random coil, beta turn, helix-loop-helix, and coiled coil conformations have also been observed (Ghosh et al., 2014; Datta et al., 2015; Verly et al., 2017). Most of the works in literature correlate the activity of these compounds to their membrane-interactions and membrane-disruptive properties (Yang et al., 2001; Bechinger and Gorr, 2017). However, a rising number of works have described the action of the peptides on interactions with intracellular components as DNA, proteins, and also some enzymes (Brogden, 2005; Azim et al., 2016).

A great variety of molecules, such as amino acids, polyamines, proteins, and peptides can be found in spider venoms, including *Lycosa erythrognatha*. In particular, the venom peptides can interact with ion channels and cell receptors, and some of them show antimicrobial activity (Santos et al., 2016). Most of the investigations on AMPs isolated from spiders have proved the activity of several of these compounds against planktonic bacteria or fungi, although many studies have also shown their activities against bacteria biofilms (Overhage et al., 2008; Ageitos et al., 2017).

Our group previously isolated and characterized a new peptide, named LyeTxI, from the venom of the spider *Lycosa erythrognatha* (Santos et al., 2010). LyeTxI shows strong antibacterial and antifungal activities, as well as a weak hemolytic activity in high concentrations. Moreover, this peptide inhibits periodontal pathogens and epithelial cells proliferation (Consuegra et al., 2013). Recently, our group showed that LyeTxI and its formulation in beta-cyclodextrin, besides being active against periodontopathic bacteria, can also be used to prevent biofilm development (Cruz Olivo et al., 2017).

Nowadays, several efforts have been made to design molecules derived from AMPs with better efficacy, low possibility to induce resistance and with less potential toxicity. Due to the great biotechnological/pharmaceutical potential of LyeTxI, its structure can be used as a template to develop new antibiotics against planktonic bacteria and fungi. Therefore, in the present work, we describe the structure and the biological activity, both *in vitro* (planktonic bacteria) and *in vivo* (a model of septic arthritis in mice), of LyeTxI-b, a peptide derived from LyeTxI. This peptide contains two structure modifications as acetylated N-terminal and a deletion of a His residue. These modifications have altered its structure and improved its activity *in vitro* and *in vivo.*

## Materials and Methods

### Peptide Synthesis and Purification

The peptide LyeTxI-b (CH_3_CO-IWLTALKFLGKNLGKLAKQQLAKL-NH_2_), was synthesized by stepwise solid-phase using the *N*-9-fluorenylmethyloxycarbonyl (Fmoc) strategy (Chan and White, 2000) on a Rink-amide resin (0.68 mmol⋅g^-1^). The following side-chain protecting groups were used: *t*-butyl for threonine, *t*-butyloxycarbonyl for lysine and tryptophan, (triphenyl)methyl for asparagine and glutamine. The couplings were performed with 1,3-diiso-propylcarbodiimide (DIC), dichloromethane in DMF for 3–4 h. Fmoc deprotection steps were carried out with piperidine/DMF (1:4; v:v) (20 min, twice). The last residue was deprotected and washed to perform the acetylation with a solution comprising 2 mL DMF, 1 mL DCM, 0.11 mol.L^-1^ DIC and 364.4 μl of 99% acetic anhydride (Sigma-Aldrich) for 40 min. The cleavage step and the side chains deprotection were performed with TFA/thioanisole/water/1,2-ethanedithiol/triisopropylsilane, (86.5/5.0/5.0/2.5/1.0, by volume) at room temperature during 3 h. The final product was precipitated with cold diisopropyl ether and lyophilized.

Two steps RP-HPLC were performed to purify the crude peptide. The first purification was done using a semi-preparative Discovery^®^ BIO Wide Pore C18 column (Supelco), previously equilibrated with 0.1% aqueous TFA (solvent A). The elution was performed with a stepped gradient of acetonitrile in 0.1% TFA (solvent B) (0–40% of solvent B in 4 min; 40–55% of solvent B in 50 min; 55–100% of solvent B in 5 min). The flow was 5.0 mL.min^-1^ and detection at 220 nm. The PepMap C18^TM^ column (4.6 mm × 150 mm) previously equilibrated with solvent A was used in the second purification. The peptide fraction was eluted with a linear gradient of solvent B (0–100% of solvent B in 30 min). The flow was 1.0 mL.min^-1^ and detection at 214 nm.

### Mass Spectrometry Analysis

The quality of peptide synthesis and purification were evaluated by MALDI-TOF/TOF mass spectrometry analyses carried out on an AutoFlex III instrument (Bruker Daltonics, Billerica, MA, United States). The samples were co-crystallized with CHCA matrix (1:1, v/v) on MTP AnchorChip 400/384 or 600/384 plates (Bruker Daltonics, Billerica, MA, United States). The instrument was operated in positive reflector mode and the results were analyzed on FlexAnalysis 3.1 (Bruker Daltonics, Billerica, MA, United States).

### Circular Dichroism Spectroscopy

The secondary structure preferences of LyeTxI-b were investigated by CD spectroscopy for the peptide in TFE:H_2_O solutions (0:100; 10:90; 20:80; 30:70; 40:60; 50:50; 60:40), in the presence of SDS micelles (detergent concentrations ranging from 0.2 to 30.0 mM), and in the presence POPC:POPG (3:1 mol:mol) phospholipid vesicles (lipid concentrations ranging from 0.05 to 2.0 mM). The spectra were recorded at 20°C on a Jasco- J-735 spectropolarimeter coupled to a Peltier Jasco PTC-423L (Tokyo, Japan). A rectangular quartz cuvette (1.0 mm path length; NSG, Farmingdale NY, United States) was used. Spectra were acquired from 260 to 190 nm using a 1.0 nm spectral bandwidth, 0.2 nm step resolution, 50 nm.min^-1^ scan speed, and 1 s response time. 6, 6, and 8 accumulations were, respectively, performed for the peptide samples prepared in TFE:H_2_O solutions, in the presence of detergent micelles and in the presence of phospholipid vesicles. Similar experiments with the respective blank solutions were also carried out for background subtraction. The peptide concentration, as determined from the tryptophan molar absorptivity (*e* = 5,550 M^-1^ cm^-1^ at 280 nm), was at 36.5 nmol.L^-1^ in all CD studies. The POPC:POPG (3:1) phospholipid vesicles were prepared as described elsewhere (Gusmao et al., 2017).

### Two-Dimensional Solution NMR Spectroscopy

Two-dimensional solution NMR experiments were carried out to determine the three-dimensional structure of LyeTxI-b. Samples were prepared by dissolving the peptide in a mixture of TFE-*d*_2_/H_2_O (60:40%, v/v) at 2.0 mM, and the pH was adjusted to 7.0 with 20 mM aqueous phosphate buffer. All NMR experiments were performed at 20°C on a Bruker Avance III spectrometer operating at a ^1^H frequency 600.043 MHz. A 5 mm triple-resonance (^1^H/^13^C/^15^N) gradient probe was used for all experiments. Water suppression was achieved by using pre-saturation. All NMR spectra were processed using NMRPipe (Delaglio et al., 1995).

Total correlation spectroscopy spectrum was acquired using the MLEV-17 pulse sequence (Bax and Davis, 1985) with a spin-lock time of 60 ms. The following parameters were used: spectral width of 6602 Hz, 512 *t*_1_ increments were collected with eight transients of 4096 points. NOESY spectra (Kumar et al., 1980) was acquired using mixing times of 80, 100, and 150 ms to check for spin diffusion. The parameters were used as follows: spectral width of 6602 Hz, 512 *t*_1_ increments were collected with 32 transients of 4096. ^1^H-^13^C HSQC spectra were acquired with F1 and F2 spectral widths of 12820 Hz and 6602 Hz, respectively. 256 *t*_1_ increments were collected with 64 transients of 1024 points. Experiments were acquired in an edited mode in such a way that CH and CH_3_ correlations showed a positive phase and CH_2_ correlations showed a negative phase (Willker et al., 1993). ^1^H-^15^N HMQC spectra were acquired with F1 and F2 spectral widths of 1824 Hz and 6602 Hz, respectively, using a fast pulse sequence (Schanda and Brutscher, 2005). 128 *t*_1_ increments were collected with 800 transients of 1024 points.

### NOE Data and Structure Calculations

The NMR spectra were analyzed using NMRView, version 5.0.3 (Johnson and Blevins, 1994). NOE intensities obtained at 150 ms mixing time were converted into 323 semi-quantitative distance restrains (190 intraresidue, 69 sequential and 64 medium range distance restrains) using the calibration described by Hyberts et al. (1992). The upper limits of the distance restrain thus obtained were 2.8, 3.4, and 5.0 Å (strong, medium, and weak NOE, respectively). Forty-four dihedral angle restrains were obtained from analysis of Cα, Hα, Cβ, N, and HN chemical shifts with the program TALOS+ (Shen et al., 2009). Structure calculations were performed using the Xplor-NIH software, version 2.27 (Schwieters et al., 2003). A total of 100 structures, starting with an extended conformation, were generated using a simulated annealing protocol, followed by 20,000 steps of simulated annealing at 1,000 K and a subsequent decrease in temperature in 15,000 steps in the first slow-cool annealing stage. The stereochemical quality of the lowest energy structures was analyzed by PROCHECK-NMR (Laskowski et al., 1996). The display, analysis, and manipulation of the three-dimensional structures were performed with the program MOLMOL (Koradi et al., 1996).

### Antimicrobial Tests

Strains of *Escherichia coli* (ATCC 25922) and *Staphylococcus aureus* (ATCC 25923) were cultured on BHI agar in aerobic conditions, while strains of *Aggregatibacter actinomycetemcomitans* (ATCC 29522) and *Streptococcus sanguinis* (ATCC 10556) were cultured on BHI in anaerobic conditions. The MIC using the microdilution method was performed to determine the bacterial susceptibility to AMPs, as previously described (Wiegand et al., 2008). Samples of LyeTxI-b were serially diluted from 91.25 to 0.17 μmol/L in MH broth and incubated with 10^5^ CFU/well for 24 h at 37°C. MIC was defined as the lowest concentration of the peptide that prevented the visible growth of the microorganism. Also, minimum bactericidal concentrations (MBCs) values were determined by plating an aliquot of MIC values by well, with no visible turbidity, in BHI agar, in aerobic conditions for *E. coli* and *S. aureus*, and in anaerobic conditions for *A. actinomycetemcomitans* and *S. sanguinis*. After 24–48 h of growth, the MBC value was determined as the lowest concentration of peptide with no visible bacterial growth on the surface of the agar. MIC and MBC assays were performed in triplicate.

### Hemolytic Assay

The hemolytic assays were performed as previously described (Santos et al., 2010), with modifications. Briefly, lamb erythrocytes were incubated with a serial concentration dilution of the peptide (91.25 μmol.L^-1^ to 0.17 μmol.L^-1^) for 1 h at 37°C. The released hemoglobin was measured using a spectrophotometry at 405 nm. Triton X-100% was used as positive control.

### Experimental Models of Arthritis

#### *Staphylococcus aureus*-Induced Arthritis

*Staphylococcus aureus* ATCC 6538 was grown in BHI agar supplement with 5% of sheep blood for 24 h. The inoculum (10^7^ CFU.mL^-1^) was injected into the tibiofemoral joint of anesthetized C57BL/6j mice as described (Amaral et al., 2016), while negative controls received saline injections. Treatments were performed directly into the joint, with LyeTxI-b (0.03 nmol), clindamycin (7.0 nmol) or saline, each starting 48 h after the bacterial injection. Seven days after the injection of the inoculum, mice were euthanized under an overdose of ketamine/xylazine anesthesia, followed by cervical dislocation, for analyses.

#### Antigen-Induced Arthritis (AIA)

C57BL/6j mice were immunized on day 1 with intradermal injection of 500 μg of mBSA (Sigma-Aldrich, St Louis, MO, United States) in 50 μL PBS emulsified in 50 μL Freund’s complete adjuvant (CFA; Sigma-Aldrich). Fourteen days later, the antigen challenge was performed by injecting 10 μg mBSA (in 10 μL sterile saline) in the right knee joint of each mouse. Mice were then intra-articularly treated with LyeTxI-b (0.03 nmol) 1 h before the challenge and 3 h after the challenge. All procedures were performed under ketamine/xylazine anesthesia, and all efforts were made to minimize animal suffering. Analyses were conducted 24 h after the challenge. Non-immunized mice were used as negative controls. Euthanasia was performed as mentioned above.

*In vivo* experiments were carried out in accordance and approved by the local Animal Experiment Ethics Committee (CETEA-UFMG; protocol no. 236/2014).

### Inflammatory Parameters Analysis

After mice euthanasia, the intra-articular cavity was exposed and washed with PBS containing 3% of bovine serum albumin (2× 5 μL). The total number of leukocytes and differential leucocytes was counted using a Neubauer chamber and cytospin preparations (Shandon III; Thermo Shandon, Frankfurt, Germany) stained with May–Grunwald–Giemsa, respectively. Then, the periarticular tissue was removed for cytokine analyses. IL-1β and CXCL1 chemokine concentrations were measured using a commercially available enzyme-linked immunosorbent assay (ELISA), following the instructions supplied by the manufacturer (R&D Systems, Minneapolis, MN, United States).

In another set of experiments, mice were euthanized for the analysis of proteoglycan loss in joint cartilage, a marker of cartilage damage. Tissue was fixed in 10% buffered formalin (pH 7.4), decalcified for 30 days in 14% EDTA. Samples were embedded in paraffin and then sectioned and stained with toluidine blue. Two sections/knee joint were microscopically examined by a single pathologist to estimate joint proteoglycan content, as previously described (Urech et al., 2010). The joint surface images of each sample were acquired and analyzed using ImageJ software (National Institutes of Health, Bethesda, MD, United States). Cartilage proteoglycan content was defined as the percentage of TB-stained area in relation to the total evaluated cartilage surface.

## Results and Discussion

### Synthesis and Liquid Chromatography

**FIGURE 1:**
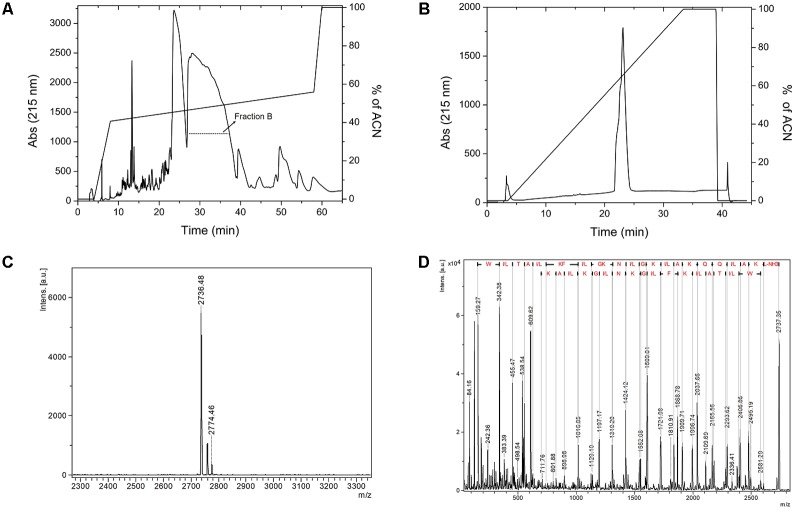
Purification and mass spectrometry analyses of LyeTxI-b synthetic peptide. **(A)** The crudely synthesized peptide was first purified by RP-HPLC using Discovery^®^ Bio Wide Pore C18^TM^ column (Supelco) with a stepped gradient of acetonitrile in 0.1% TFA. **(B)** Purification of fraction B by RP-HPLC on PepMap C18 column (4.6 mm × 150 mm). The column was previously equilibrated with 0.1% aqueous TFA and the peptide eluted with a linear gradient of acetonitrile in 0.1% TFA. **(C)** LyeTxI-b was analyzed by MALDI-TOF-TOF mass spectrometry and **(D)** the primary sequence was confirmed by MS/MS using LIFT fragmentation.

After chemical synthesis, we purified the peptide using two-step RP-HPLC. The first RP-HPLC assay on Discovery^®^ BIO Wide Pore C18 column (**Figure 1A**) shows two major fractions eluted with an acetonitrile gradient (50–55% of solvent), named fraction A and fraction B. Only fraction B was submitted to another RP-HPLC experiment on a PepMap C18^TM^ (**Figure 1B**), and the relative purity of the fraction was analyzed by MALDI-TOF/TOF mass spectrometry. The peptide of interest was eluted in fraction B, which was evidenced by a higher *m/z* intensity 2736.4, corresponding to the expected mass of the N-terminally acetylated peptide (MW 2737.3 Da) (**Figure 1C**). The peptide was therefore named LyeTxI-b. To confirm the primary structure of LyeTxI-b, this compound was subjected to MS/MS fragmentation (**Figure 1D**).

### Circular Dichroism Spectroscopy

**FIGURE 2:**
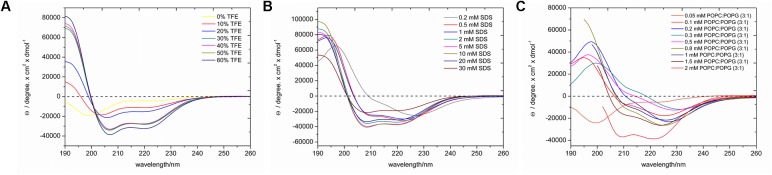
CD spectra of LyeTxI-b in the presence of **(A)** TFE:H_2_O solutions, **(B)** SDS micelles, and **(C)** POPC:POPG (3:1) vesicles. At higher phospholipid concentrations light scattering artifacts strongly increase at short wavelengths (≤203 nm) and only the spectral components used for the analysis are shown in **(C)**.

LyeTxI-b exhibits random coil conformations in the aqueous environment, as evidenced by the minimum near 200 nm in **Figure 2A**. Upon addition of TFE, the peptide adopts a helical conformation, as noticed by the appearance of two minima near 208 and 222 nm and a maximum near 192 nm (**Figure 2A**), which show relatively small intensities up to 20% of TFE, however, significant increases are observed in solutions containing 30–50% of TFE, which present very similar spectral profiles. This is a very common feature of linear cationic AMPs peptides, which show no conformational preferences in aqueous environments, whereas well-defined conformations are observed in the presence of organic co-solvents or other membrane mimetic environments (Gusmao et al., 2017). Interestingly the spectrum obtained for the peptide in the presence of 60% of TFE is consistent with even higher helical contents. Some investigations using small angle X-ray and dynamic light scattering of different alcohol–water solutions have indicated the formation local clusters, which reach maximum size at about 40% of the co-solvent in the case of TFE:H_2_O mixtures, whereas higher concentrations of TFE lead to smaller size clusters due to a higher homogeneity of the respective solutions (Kuprin et al., 1995; Gast et al., 2001; Othon et al., 2009). The larger aggregates may reduce the local polarity near the peptide and induce intramolecular bond formation or peptide aggregation, which can be correlated to differences in helicity. Since a higher helicity is observed in the presence of 60% of TFE and to avoid complications due to the formation of local aggregates, we decided to investigate in atomic detail the three-dimensional structure of LyeTxI-b by NMR spectroscopy in a solution containing 60% of the co-solvent (see below).

In order to confirm the helical conformation in membrane mimetic environments, the secondary structural preferences were also investigated in the presence of SDS micelles and POPC:POPG (3:1) vesicles and spectra with helical profiles were observed in both cases. In the presence of the micellar detergent (**Figure 2B**), the intensities of the characteristic maximum and minima increase with the SDS concentration until a plateau (5 mM of SDS) is reached. In the presence of vesicles (**Figure 2C**), the minimum and maxima intensities also increase with the phospholipid concentration, however, significant augments in the two minima intensities are observed at 2 mM of phospholipids. These results indicate that the peptide adopts well-defined helical conformations in the presence of phospholipid vesicles, however, a membrane-bound (helical) and a water-soluble (random coil) states coexist at smaller phospholipid to peptide ratios, as previously observed for other AMPs (Voievoda et al., 2015).

### Two-Dimensional Solution NMR Spectroscopy

**FIGURE 3:**
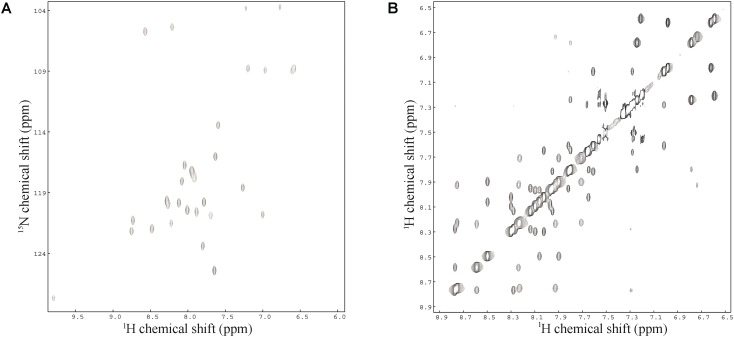
**(A)** ^1^H-^15^N HMQC spectrum and **(B)** amide-amide region of the NOESY spectrum of LyeTxI-b at 2.0 mM in TFE-*d*_2_:H_2_O (40:60) – pH 7.0 (phosphate buffer), 20.0°C.

**FIGURE 4:**
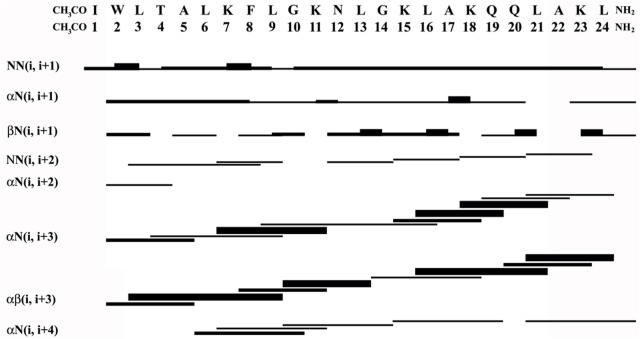
Graphical summary of NOE correlations characteristic of helical structures observed in the NOESY spectrum of LyeTxI-b.

**FIGURE 5:**
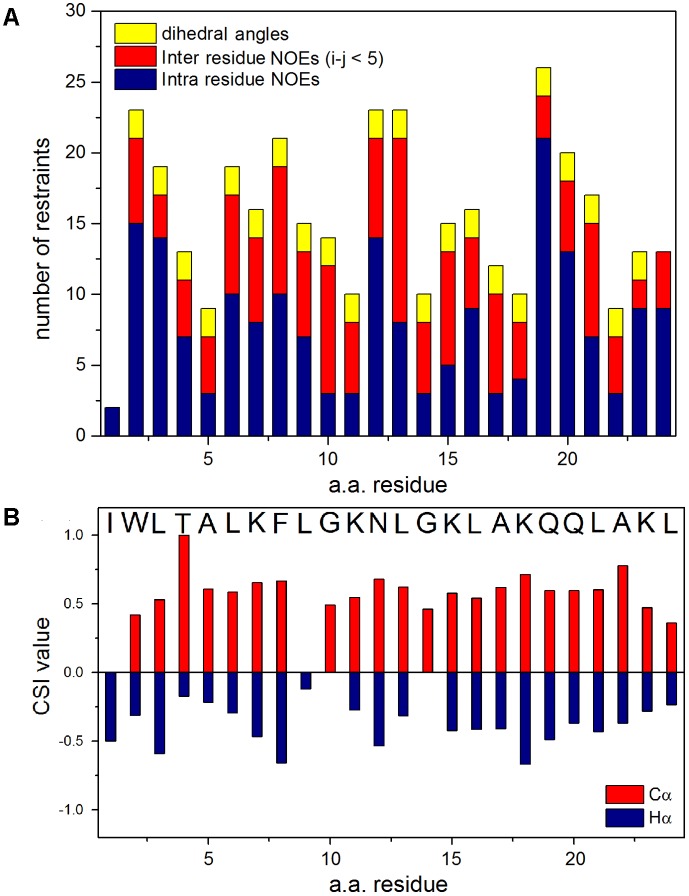
**(A)** Distribution of distance and dihedral angle restraints per residue used in the structural calculations for LyeTx-b peptide. Number of intra-residue NOE restraints is shown in blue, the number of inter-residue NOE restraints (i–j < 5) are shown in red and the number of dihedral angle restraints in yellow. **(B)** Values of Chemical-Shift Index (CSI) derived from the Cα (red) and Hα (blue) resonances.

**FIGURE 6:**
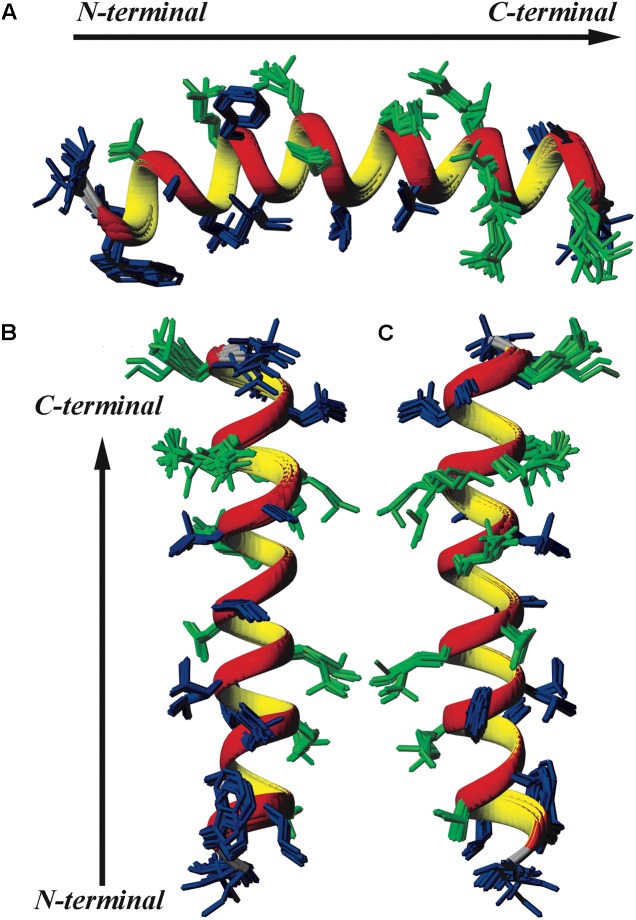
Superposition of the 10 lowest energy structures of LyeTxI-b. Hydrophilic residues are shown in green and hydrophobic residues in dark blue. **(A)** Represents a horizontal perspective of the peptide helix, whereas **(B)** highlights a face of the helix composed mostly of hydrophobic residues and **(C)** a face composed mostly of hydrophilic and charged residues.

Sequence-specific assignments have been performed from simultaneous analysis of NMR contour maps, as previously indicated (Resende et al., 2008). The dispersion of chemical shifts observed in the ^1^H-^15^N HMQC spectrum, as well as the high number of cross-peaks detected in the NOESY contour map (**Figure 3**) indicate a well-folded peptide conformation. **Figure 4** presents the summary of through-space correlations that characterize peptide secondary structure, obtained from NOESY. In accordance with the data obtained from CD spectroscopy at similar conditions, several NN (i, i+1), αN (i, i+3), αβ (i, i+3) and αN (i, i+4) connections indicate that the peptide presents a well-defined helical segment from Trp-2 to the last amino acid residue, including the C-terminal carboxamide, which is involved in an αN (i, i+4) interaction. The distribution of distance and dihedral angle restraints per residue are presented in **Figure 5A**. This trend of α-helix secondary structure is also confirmed by the Hα and Cα chemical shift indexes (CSI) (Wishart et al., 1992; Tremblay et al., 2010), as presented in the **Figure 5B**. **Figure 6** presents the overlap of the 10 most stable structures obtained after simulated annealing protocol. This set of structures showed RMSD values of 0.98 and 0.46 Å for the superposition of all heavy and of the backbone atoms, respectively, and all of the *ϕ* and *ψ* angular pairs are located in the most favorable regions of the Ramachandran Plot (data from PROCHECK NMR), indicating the good quality of the structural model obtained.

Similarly to LyeTxI (Santos et al., 2010), LyeTxI-b α-helix does not show a clear partition between hydrophobic and hydrophilic faces, which is observed for many other antibiotic peptides, such as phylloseptins and dermadistinctin K (Resende et al., 2008; Verly et al., 2009). However, LyeTxI-b presents a higher structural stability near the N-terminus when compared to the native peptide LyeTxI, since LyeTxI-b helix is defined by a greater number of medium-range NOEs near the N-terminus, especially medium and strong-intensity αβ (i, i+3) correlations. The higher structural stability near LyeTxI-b N-terminus is certainly related to the terminal amine acetylation, which eliminates the positive charge and stabilizes the positive end of the helix dipole. The acetylation also allows extra hydrogen bond interactions involving the acetyl carbonyl group and the amidic hydrogens near N-terminus. Extra hydrogen bonds interactions, as well as helix dipole neutralization properties due to either C-terminal carboxyamidation or N-terminus acetylation, are known to promote structural stability in helical segments (Resende et al., 2008; Zanin et al., 2016). An interesting feature observed for the LyeTxI-b structure is a helix curvature (**Figure 3A**), which is correlated to the NN (i, i+2) NOE correlations involving several residues of the helical segment. This sort of NOEs was observed for the native peptide with a minor proportion, inducing a less pronounced curvature of the peptide helix (Santos et al., 2010).

Another example of an AMP that does not show an amphipathic partition is the Htr-M, in which a lysine residue represents a discontinuity within the helix hydrophobic face. This discontinuity has an important structural role in the homodimeric peptide homotarsinin (Htr), since the positively charged side chain of this lysine residue interacts with a negatively charged aspartate residue and a hydrophilic serine residue of the other peptide chain. These electrostatic interactions are important to stabilize the homodimer coiled coil three-dimensional structure (Verly et al., 2017).

### Antimicrobial Activity in Planktonic Culture

LyeTxI-b showed a potent antimicrobial inhibition activity against Gram-positive and Gram-negative planktonic bacteria under aerobic and anaerobic conditions (**Table 1**). We observed that LyeTxI-b was exceptionally active against *E. coli* and *S. aureus*. The MBC values were the same as those obtained for MIC in all tests. The topic antibiotic chlorhexidine acetate was used as a positive control.

**Table 1:** Minimum inhibitory concentration (MIC) and minimum bactericidal concentration (MBC) of LyeTxI-b and chlorhexidine acetate.

	*E. coli*		*S. aureus*		*A. actinomycetemcomitans*		*S. sanguinis*	
	MIC	MBC	MIC	MBC	MIC	MBC	MIC	MBC
Compound	μmol.L^-1^	μmol.L^-1^	μmol.L^-1^	μmol.L^-1^	μmol.L^-1^	μmol.L^-1^	μmol.L^-1^	μmol.L^-1^
LyeTxI	7.81^∗∗^	–	–	–	10.6^∗^	20.12^∗^	10.9^∗∗∗^	21.8^∗∗∗^
LyeTxI-b	0.71	0.71	2.85	2.85	11.4	11.4	5.7	5.7
Chlorhexidine acetate	125	–	31	–	125	–	62.5	–

*Data of LyeTxI were obtained from (Consuegra et al., 2013) ^∗^; (Santos et al., 2010) ^∗∗^; (Cruz Olivo et al., 2017) ^∗∗∗^ and correspond to the same strain references used to LyeTxI-b.*

The MIC value of LyeTxI-b for *E. coli* was 10-fold higher when compared to that of the LyeTxI peptide (Santos et al., 2010). On the other hand, the activity of LyeTxI-b for *S. aureus* was similar to the previously observed for the native peptide (Santos et al., 2010). Moreover, LyeTxI-b was able to decreased around 50% of MBC but with similar MIC in *A. actinomycetemcomitans*, when compared to the original peptide in the work of Consuegra et al. (2013). It is known that the lipid composition of the plasma membrane considerably affects the action of AMPs (to review Malanovic and Lohner, 2016; Malmsten, 2016). Gram-negative bacteria, in their majority, present a higher concentration of the zwitterionic lipid phosphatidylethanolamine when compared with Gram-positive bacteria (Epand and Epand, 2011).

In general, LyeTxI-b has antimicrobial activity at μmol concentrations, similarly to previously described AMPs, such as magainins, LL-37 and others (Park et al., 2009; Joshi et al., 2010; Brogden and Brogden, 2011; Haisma et al., 2014; Ageitos et al., 2017).

### Hemolytic Assays

**FIGURE 7:**
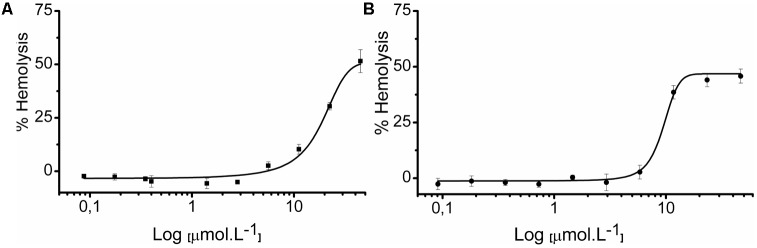
Hemolytic activity of LyeTxI and LyeTxI-b. Lamb erythrocytes suspended in PBS were incubated for 1 h with increasing concentrations from 0.35 to 44.8 μmol.L^-1^ and 0.36 to 46.8 μmol.L^-1^, of **(A)** LyeTxI and **(B)** LyeTxI-b, respectively. The control of 100% of cell lysis was considered by hemoglobin release in the presence of Triton X-100 (1% by volume). Hemoglobin release was measured at 405 nm. ED_50_: LyeTxI = 42.54 μmol.L^-1^ while LyeTxI-b at 46.8 μmol.L^-1^ had 46.83% of hemolysis.

Our group previously demonstrated the hemolytic capacity of LyeTxI, with an ED_50_ of 130 μM on rabbit erythrocytes (Santos et al., 2010). Therefore, we also tested the hemolytic activity of LyeTxI-b, to evaluate if the loss of an amino acid residue and the N-terminus acetylation would modify this property. In this work, we performed the assay on lamb erythrocytes, for both peptides. As shown in **Figure 7**, LyeTxI had an ED_50_ value of 42.54 μmol.L^-1^, while LyeTxI-b showed approximately 46.83% of hemolysis at 46.6 μmol.L^-1^. This result indicates a slight increase in LyeTxI-b activity on eukaryotic cells, which is not significant, considering its 10-fold increase in bactericidal capacity for *E. coli*. Moreover, we observed a high level of hemolysis for LyeTxI in lamb erythrocytes, when compared to rabbits. It is known that mammalian species differ in their erythrocyte susceptibility, and lamb erythrocytes have been considered more fragile than those from rabbit (Matsuzawa and Ikarashi, 1979). In addition, it may be pointed out that in recent results obtained by our group (not shown) LyTxI-b showed only mild cytotoxicity against lung and kidney normal cell lines (Abdel-Salam et al., work in submission).

### LyeTxI-b Controls Bacterial Growth and Tissue Inflammation *in Vivo*

Considering our positive results regarding LyeTxI-b activity in the MIC assay, we evaluated the effectiveness of this peptide in a model of *S. aureus*-induced arthritis in mice (Boff et al., 2018). Initially, we determined the MIC for *S. aureus* ATCC 6538, since the strain used in this model was different from the previous MIC assay. The MIC value for LyeTxI-b was 2.85 μmol.L^-1^, while for clindamycin it was 367 μmol.L^-1^ (control). It is noteworthy that in the growth inhibition tests we used 10^5^ CFU.mL^-1^ while the SA model was established with 10^7^ CFU.mL^-1^. Therefore, the MIC concentrations used was two-fold higher for the *in vivo* test. Thus, for each 10 μL injection in treatment models, 80 ng of LyeTxI-b and 3120 μg of clindamycin were applied.

**FIGURE 8:**
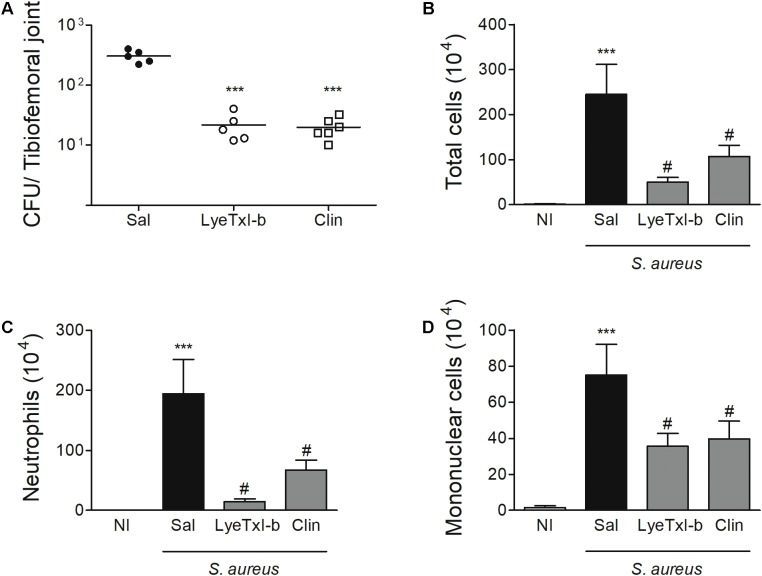
LyeTxI-b decreases bacteria load and cellular recruitment to the joint. The arthritis was induced by intra-articular injection of *S. aureus* (10^7^ CFU/10 μL) in mice. The treatment with LyeTxI-b (0.03 nmol) and clindamycin, Clin (7 nmol) by ipsilateral joint injection was performed every other day, starting on day 1, after arthritis induction. The analyses were performed 7 days after *S. aureus* injection for **(A)** bacterial load in whole joint and, **(B)** total cells, **(C)** neutrophils and **(D)** mononuclear cells accumulated into the joint cavity. ^∗∗∗^*p* < 0.05 compared to non-infected mice; ^#^*p* < 0.05 compared to saline-treated infected mice. *n* = 6–8 mice per group. CFU, colony-forming unit (definir); Sal, saline; NI, non-infected mice.

*Staphylococcus aureus*-induced joint inflammation is associated with a massive recruitment of neutrophils to the joint that, together with the presence of bacteria, cause articular damage and pain (Mathews et al., 2010). In this model, a high concentration of bacteria can be detected in the joint 7 days after the challenge, and the treatment with LyeTxI-b reduced this number (**Figure 8A**). Furthermore, LyeTxI-b reduces cell accumulation in *S. aureus*-challenged joint, including the number of total cells, neutrophils and mononuclear cells (**Figures 8B–D**). Therefore, we can speculate that both treatments, using LyeTxI-b and clindamycin, were able to decrease the pain symptom, since the pain process can be associated with the mediators released by neutrophils (Sachs et al., 2011), as well as the direct activation of nociceptors by *S. aureus* (Chiu et al., 2013). It is worth mentioning that the treatment with the peptide decreased the bacterial load to the same level as the antibiotic treatment, not showing significant difference between the groups in the experimental model of SA. However, the therapeutic concentration of our peptide was more than two hundred lower (0.03 nmol) compared to that of the antibiotic (7 nmol), indicating its higher potency.

**FIGURE 9:**
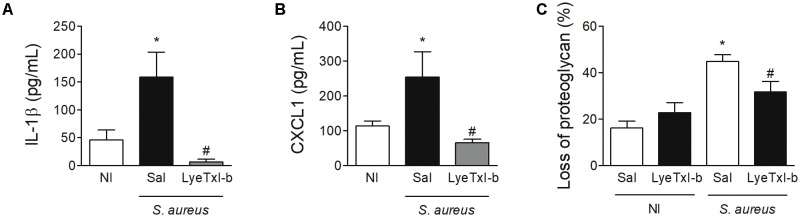
LyeTxI-b treatment decreased *S. aureus*-induced joint inflammation. After arthritis induction by intra-articular injection of *S. aureus* (10^7^ CFU/10 μL) in mice, the animals were treated with LyeTxI-b (0.03 nmol) and clindamycin (7 nmol) by ipsilateral joint injection every other day. Seven days after *S. aureus* injection, the levels of **(A)** IL-1β and **(B)** CXCL1 in periarticular tissue were measured by ELISA while the loss of proteoglycan was determined by toluidine blue stain **(C)**. ^∗^*p* < 0.05 compared to non-infected mice; ^#^*p* < 0.05 compared to saline-treated infected mice. *N* = 6–8 mice per group, NI, non-infected mice.

Similarly, the treatment with LyeTxI-b and clindamycin also reduced the level of IL-1β pro-inflammatory cytokine and CXCL1chemokine in the joint, when compared to non-treated joints (**Figures 9A,B**). IL-1β has been shown as an important factor in the pathogenesis of septic arthritis in an experimental model of intravenous injection (Hultgren et al., 2002) and CXCL1 is essential for the recruitment of neutrophils (Lira et al., 1994). Another important characteristic of septic arthritis is tissue damage. Here, the injection of *S. aureus* caused an important loss of proteoglycans on articular cartilages while the treatment with LyeTxI-b preserved the integrity of the joint (**Figure 9C**). Importantly, the injection of LyeTxI-b in naïve joint did not cause tissue inflammation.

Our results show that LyeTxI-b-treated mice present decreased bacteria load and inflammation in the joint similar to clindamycin but in different concentration, as mentioned above. Since the presence of bacteria in a joint is sufficient to stimulate resident cells to produce pro-inflammatory mediators and cell recruitment, the reduced number of bacteria in LyeTxI-b-treated group could be enough to reduce all other inflammatory markers, as observed in **Figures 8**, **9**. Thus, we questioned whether LyeTxI-b could have a direct role in the reduction of joint inflammation, independently on its bactericidal/bacteriostatic property. To answer this question, we used an experimental model of immunization-induced arthritis (without infection – AIA model). The treatment with LyeTxI-b did not alter the recruitment of neutrophils to the joint, which are important markers of tissue inflammation in this model (AIA: 146.08 ± 15.87 – *n* = 5, AIA + LyeTxI_b: 162.28 ± 12.71 – *n* = 6; mean ± SEM). Thus, this result showed a non-direct role of LyeTxI-b on joint inflammation, and the reduction of *S. aureus*-induced arthritis seems to be caused by the control of bacterial growth in the joint. In sum, the ability of LyeTxI-b to control bacterial growth in the joint is sufficient to reduce the main inflammatory markers of SA model. In addition, preliminary nociceptive assays using an electronic version of von Frey test with LyeTxI-b in mice did not show any effect (data not shown).

Since the treatment of SA patients mostly involves use of antibiotics and considering that an increased number of patients infected with bacteria are resistant to antibiotics, the development of new therapeutic options became essential for a better control of infection and tissue damage (Weston et al., 1999; Sharff et al., 2013). Thus, LyeTxI-b is a prominent model for the development of new antibiotics.

## Conclusion

In the present work, we characterized LyeTxI-b, a peptide derived from the spider toxin LyeTxI, with two modifications: removal of a single amino acid residue and N-terminus acetylation. We show that these small alterations are able to improve the antibiotic activity of the peptide. This is also observed in the organisms under study, i.e., different strains of the same microorganism or different species may have different susceptibilities to the drug. Therefore, our results contribute to the emerging area of antibiotics derived from AMPs, as a new alternative for the treatment of infections that are resistant to conventional drugs.

## Author Contributions

PR synthesized and purified the peptide and performed MALDI-TOF and CD analyses. RV and JR performed the NMR experiments and analyzed the CD and NMR data. PR and MC investigated the study on MIC. PR and MM-B investigated the study on hemolysis. DB and FA investigated the study on *in vivo* model. PR and MdL wrote the original draft. PR, MM-B, MC, AP, FA, JR, and MdL wrote, reviewed, and edited the manuscript. MdL envisioned the concept. DS, AP, and MdL supervised the study.

## Conflict of Interest Statement

The authors declare that the research was conducted in the absence of any commercial or financial relationships that could be construed as a potential conflict of interest.

## Abbreviations

AMPs: antimicrobial peptides
BHI: brain heart infusion
CD: circular dichroism
CFU: colony-forming unit
CHCA: α-cyano-4-hydroxycinnamic acid
Clin: clindamycin
CXCL1: chemokine (C-X-C motif) ligand 1
DMF: *N*,*N*-dimethyl-formamide
ED_50_: effective dose 50
^1^H-^15^N HMQC: ^1^H-^15^N heteronuclear multiple quantum coherence
^1^H-^13^C HSQC: ^1^H-^13^C Heteronuclear Single Quantum Coherence
Htr: homodimeric peptide homotarsinin
Htr-M: homotarsinin monomeric chain
IL-1β: interleukin 1 beta
NMR: nuclear magnetic resonance
NOESY: nuclear Overhauser enhancement spectroscopy
MALDI-TOF/TOF: matrix-assisted laser desorption/ionization – time of flight
MBC: minimal bactericidal concentration
mBSA: methylated bovine serum albumin
MH: Mueller-Hinton
MIC: minimum inhibitory concentration
PBS: phosphate buffered saline
POPC: 1-palmitoyl-2-oleoyl-*sn*-glycero-3-phosphocholine
POPG: 1-palmitoyl-2-oleoyl-phosphatidylglycerol
RP-HPLC: reverse phase high-performance liquid chromatography
SDS: sodium dodecyl sulfate
TFE-*d*_2_: 2,2,2-trifluoroethanol-1,1-*d*_2_
TOCSY: total correlation spectroscopy
